# Airway Smooth Muscle Regulated by Oxidative Stress in COPD

**DOI:** 10.3390/antiox12010142

**Published:** 2023-01-06

**Authors:** Hiroaki Kume, Ryuki Yamada, Yuki Sato, Ryuichi Togawa

**Affiliations:** Department of Infectious Diseases and Respiratory Medicine, Fukushima Medical University Aizu Medical Center, 21-2 Maeda, Tanisawa, Kawahigashi, Aizuwakamatsu City 969-3492, Fukushima, Japan

**Keywords:** oxidants, antioxidants, Ca^2+^ dynamics, Ca^2+^ sensitization, tracheal smooth muscle, phenotype changes, reactive oxygen species

## Abstract

Since COPD is a heterogeneous disease, a specific anti-inflammatory therapy for this disease has not been established yet. Oxidative stress is recognized as a major predisposing factor to COPD related inflammatory responses, resulting in pathological features of small airway fibrosis and emphysema. However, little is known about effects of oxidative stress on airway smooth muscle. Cigarette smoke increases intracellular Ca^2+^ concentration and enhances response to muscarinic agonists in human airway smooth muscle. Cigarette smoke also enhances proliferation of these cells with altered mitochondrial protein. Hydrogen peroxide and 8-isoprostans are increased in the exhaled breath condensate in COPD. These endogenous oxidants cause contraction of tracheal smooth muscle with Ca^2+^ dynamics through Ca^2+^ channels and with Ca^2+^ sensitization through Rho-kinase. TNF-α and growth factors potentiate proliferation of these cells by synthesis of ROS. Oxidative stress can alter the function of airway smooth muscle through Ca^2+^ signaling. These phenotype changes are associated with manifestations (dyspnea, wheezing) and pathophysiology (airflow limitation, airway remodeling, airway hyperresponsiveness). Therefore, airway smooth muscle is a therapeutic target against COPD; oxidative stress should be included in treatable traits for COPD to advance precision medicine. Research into Ca^2+^ signaling related to ROS may contribute to the development of a novel agent for COPD.

## 1. Introduction

**Figure 1:**
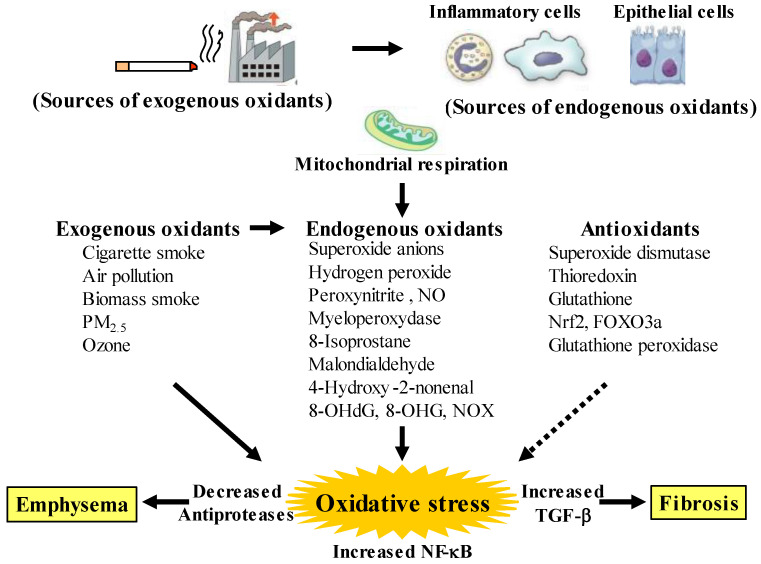
Oxidants and antioxidants involved in COPD, and relationships between oxidative stress and the pathology related to this disease. Oxidative stress in the lungs results from increased exogenous and endogenous oxidants, and from reduced antioxidants. Endogenous oxidants are generated by mitochondrial respiration. Elevated production of endogenous oxidants continues after stopping smoking. Increased oxidative stress is caused by a lack of balance between oxidants and antioxidants. Exogenous oxidants are derived from cigarette smoke, air pollution and biomass smoke, etc.; endogenous oxidants are derived from inflammatory cells (macrophages, neutrophils) and airway epithelial cells. Oxidative stress results in emphysema in alveolar areas with decreased antiproteases, and in fibrosis in the small airways with increased transforming growth factor (TGF)-β. PM: small particulate matter, 8-OHdG: 8-hydroxy-2′-deoxyguanosine, 8-OHG: 8-oxo-7,8-dihydroguanosine, NOX: membrane-bound NADPH oxidases, Nrf2: nuclear erythroid-2 related factor 2, FOXO3a: forkhead box O3a. Arrows: activation, dotted arrows: inactivation.

**Figure 2:**
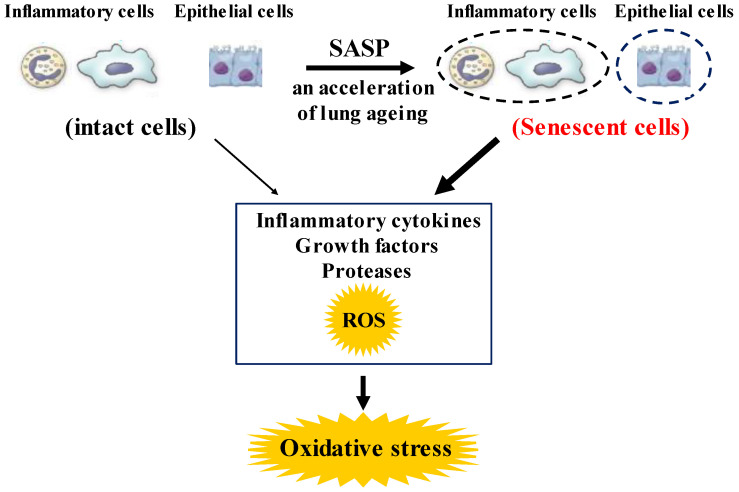
Roles of senescence in inflammatory and airway epithelial cells to enhance oxidative stress in COPD. These senescent cells in the lungs synthesize inflammatory cytokines, growth factors proteases, and ROS more than intact cells in them, referred to as senescence-associated secretary phenotype (SASP). These phenotype changes in these cells perhaps potentiate not only the lung inflammation but also oxidative stress in COPD. ROS: Reactive oxygen species.

Although chronic obstructive pulmonary disease (COPD) is simply diagnosed based on persistent air flow limitation that will not return to the normal range, using spirometric measurements, this disease is heterogenous and complex in symptoms, disease progression, lung function and response to therapies [1]. The pathogenesis of this disease results from chronic lung inflammation due to cigarette smoke and other environmental exposures (biomass fuel etc.); and this chronic inflammation is associated with activation not only of neutrophils and macrophages but also of eosinophils. While these responses to lung inflammation are normal in many healthy subjects, in contrast, the response is potentiated in patients who develop COPD. This chronic lung inflammation affects distal airways, leading to emphysema and small airway fibrosis (pathological characteristics of this disease) [2,3]; and these pathological alterations in COPD are progressive in most cases [1]. The mechanisms of this modified inflammation are not understood well. Moreover, a wide variety of inflammatory mediators are related to this chronic lung inflammation. For this reason, specific treatment for inflammation is not well established in this disease. Oxidative stress is defined as a state in which oxidation exceeds the capacity of antioxidant systems in the body secondary to a loss of the balance between them. Disturbances in the normal redox state of cells can cause toxic effects through the production of peroxides and free radicals that damage all components of the cell, including proteins, lipids, and DNA. Oxidative stress from oxidative metabolism causes base damage, as well as strand breaks in DNA. Base damage is mostly indirect and caused by reactive oxygen species (ROS) generation, e.g., O_2_^•−^ (superoxide anion radical), ^•^OH (hydroxyl radical), H_2_O_2_ (hydrogen peroxide) and O_3_ (ozone) (Figure 1) [4]. Inflammatory cells as described above are recruited into the lungs in COPD [5]; and structural cells in the respiratory system (airway epithelial cells, fibroblasts, and endothelial cells) also contribute to the lung inflammation. These cells generate multiple mediators, including cytokines that perpetuate and amplify the inflammation in the lungs. These cells are also important sources of ROS, leading to oxidative stress in the lungs (Figure 1). Oxidative stress in the lungs due to exogenous oxidants (cigarette smoke, biomass fuel, air pollution) and endogenous oxidants (ROS generated by inflammatory cells, epithelium) are associated with clinical and pathophysiological characteristics of COPD (Figure 1) [6]. Mitochondrial respiration is an important source of ROS, and cigarette smoke produces excessive ROS via mitochondrial dysfunction (Figure 1) [7]. It is now generally considered that COPD results from an acceleration of lung ageing with the accumulation of senescent cells [8,9,10]. Senescent cells secrete high levels of inflammatory cytokines, immune modulators, growth factors, and proteases, referred to as senescence-associated secretory phenotype (SASP) [10]. This phenotype change is perhaps an essential mechanism in the chronic lung inflammation of COPD [11]. Since senescent cells also release ROS more than intact cells, this chronic lung inflammation potentiates oxidative stress in COPD (Figure 2). Therefore, oxidative stress is probably a major driving mechanism of many of the pathophysiological changes in COPD [12]. 

To improve the management and treatment for COPD, patients with COPD should be classified by grouping according to distinct clinical phenotypes. These groupings, based on multiple dimensions (clinical, physiological, imaging, and endotyping) determine clusters of patients with common characteristics, which are associated with clinically meaningful outcomes such as symptoms, exacerbations, response to therapy, and disease progression (stratified medicine). Moreover, since several phenotypes can coexist in individual patients with COPD, an approach due to therapeutic target identified phenotypes and endotypes (treatable traits) has been proposed as an advanced therapy recently (precision medicine) [13]. Although oxidative stress perhaps plays an important role in amplifying the chronic lung inflammation in COPD [14], little is currently known about the involvement of oxidative stress in the pathogenesis of COPD. Therefore, research for clinical phenotype classification focused on oxidative stress is needed to establish precision medicine for development of the therapeutic management for COPD. 

In this chapter, roles of oxidative stress on airway smooth muscle are described with fucuses on functional alterations that bring about phenotype changes related to the characteristic pathophysiology in COPD. Involvement of Ca^2+^ signaling due to Ca^2+^ dynamics and Ca^2+^ sensitization is also reviewed as a mechanism of the functional alterations in airway smooth muscle induced by oxidative stress in COPD.

## 2. Oxidative Stress in COPD

### 2.1. Pathological Features 

Oxidative stress occurs in the lungs during COPD, leading to characteristic pathological changes in this disease (Figure 1). It is well proven by data derived from bronchial biopsy [15], sputum examination [16], and in vitro studies [17] that inflammatory cells such as neutrophils, macrophages and T lymphocytes infiltrate and various proinflammatory molecules are present at increased levels in smokers’ lungs. Inflammatory cells, particularly neutrophils and macrophages that are recruited into the lungs, as well as structural cells, such as airway epithelial cells and fibroblasts, generate endogenous oxidants (ROS) in the lungs, leading to destruction of peripheral airways and alveoli. Mitochondrial respiration in these related cells is a key source of ROS, and cigarette smoke enhances generation of ROS through mitochondrial dysfunction, supporting the pathophysiological characteristics in COPD [18,19]. These destructive processes overcome the local protective mechanisms, and cause tissue damage without manifestations. The inflammatory tissue damage may be perpetuated for a long time after smoking cessation in patients with COPD [20]. Cigarette smoke causes the chronic lung inflammation; however, only about 20% of smokers develop COPD, indicating that there are factors that increase susceptibility and amplify the normal inflammatory response to cigarette smoke. Although these mechanisms are still unknown in detail, this phenomenon is probably involved in oxidative stress due to synthesis of ROS and imbalance of local proteolysis/antiproteolysis states that are related to oxidative stress (imbalance of oxidants/antioxidants). 

### 2.2. Oxidants Related to COPD 

Oxidative stress is recognized as a major predisposing factor of the inflammatory response related to COPD. Oxidative stress is probably associated with the pathology and severity of COPD. Oxidative stress is potentiated in patients with COPD, especially when acute exacerbations occur. Cigarette smoke, air pollution and biomass smoke are major exogenous oxidants related to COPD in the lungs, referred to as exogenous oxidative stress, but oxidative stress also arises from endogenous processes due to endogenous oxidants, after stop smoking, referred to as endogenous oxidative stress (Figure 1). The number of activated alveolar macrophages is markedly increased in the lungs of patients with COPD, compared to healthy subjects; and a large amount of ROS is released from these activated macrophages as superoxide anions and hydrogen peroxide (H_2_O_2_) [21]. This phenomenon is more potentiated during COPD exacerbations. Activated neutrophils also infiltrate to the lungs in patients with COPD, and activated neutrophils release a large amount of ROS, especially during COPD exacerbations [22]. In patients with COPD, generation of 4-hydroxy-2-nonenal (4HNE) is increased in the lungs, indicating that lipid peroxidation, a marker of oxidative stress, occurs on endogenous lipids [23]. Clinical studies have demonstrated that H_2_O_2_, 8-isoprostane, 4HNE, myeloperoxidase (MPO) and malondialdehyde (MDA) (endogenous oxidants as biomarkers of oxidative stress) are increased in exhaled breath condensate in patients with COPD [13,24,25,26,27], compared to healthy individuals; and these makers are more elevated during exacerbations [28]. These markers, such as MDA, 8-isoprostane, 8-hydroxy-2′-deoxyguanosine (8-OHdG) and MPO, are also elevated in sputum from patients with COPD [29,30]. Furthermore, nucleic acid oxidation, 8-oxo-7,8-dihydroguanosine (8-OHG) in RNA and 8-OHdG in DNA are elevated in alveolar lung fibroblasts from patients with emphysematous COPD [18,31]. These augmented biomarkers of oxidative stress do not decrease, and remain elevated in ex-smokers after the cessation of smoking, suggesting that persistent lung inflammation is caused by endogenous oxidative stress [25]. 

The respiratory system is constantly exposed to oxidative stress due to sources of endogenous ROS generated by mitochondrial respiration and inflammatory responses to bacterial and viral infections. The persistent oxidative stress in COPD results not only from activated neutrophils and macrophages but also from epithelial cells in the respiratory system. Oxidative stress is associated with mitochondrial respiration in these structural cells [32]. Other sources of intracellular ROS include the cytoplasmic ROS generating enzymes, such as membrane-bound nicotinamide adenine dinucleotide phosphate (NADPH) oxidases (NOX) and the xanthine/xanthine oxidase system, as well as neutrophil derived MPO [33]. Superoxide anions, relatively weak oxidizing agents, are mainly produced endogenously by NOX; and are rapidly converted to more damaging ROS, such as the hydroxyl radical and H_2_O_2_, or the powerful and damaging peroxynitrite radical in the presence of nitric oxide (NO) [34]. MPO is released from activated neutrophils which are recruited into the lungs of patients with COPD; MPO also produces very destructive hypochlorous acid such as 3-chlorotyrosine [35]. However, in healthy adults, intracellular antioxidant defenses can overcome these damaging ROS, thus limiting their cellular effects; on the other hand, in patients with COPD these antioxidant defenses are overwhelmed. Increased oxidative stress has great effects on driving the pathophysiology of COPD as described below [33]. Oxidative stress causes activation of the proinflammatory transcription factor nuclear factor-KB (NF-κB) pathways; expression of NF-κB is augmented in COPD in airway epithelium and macrophages in patients with COPD. Oxidative stress also causes activation of the transforming growth factor (TGF)-β1 pathways, which acts on the lung epithelium, and induces fibrotic repair via driving epithelial-to-mesenchymal transition (EMT) [36], leading to small airway fibrosis. The inhibitory effects of TGF-β1 on nuclear erythroid-2 related factor 2 (Nrf2) bring about reduced expression of endogenous antioxidants [37]. Oxidative stress increases the expression of matrix metallopeptidase 9 (MMP9), an elastolytic enzyme, related to the development of emphysema.

### 2.3. Antioxidants Related to COPD 

Increased oxidative stress may be potentiated with a reduction in endogenous anti-oxidant-induced defenses in patients with COPD (Figure 1). A clinical trial has demonstrated that concentrations of glutathione are lower in bronchoalveolar lavage fluid from unstable COPD patients with frequent exacerbations than in that from stable COPD [38]. Extracellular superoxide dismutase (SOD3) is decreased around small airways in patients with COPD [39]. Thioredoxin, which is an important regulator of redox balance, is decreased in COPD [38]. Nrf2 and forkhead box O3a (FOXO3a) are decreased in the lungs of patients with COPD [40,41]. Nrf2 and FOXO3a, which are key transcription factors that regulate multiple antioxidant genes, protect the respiratory system against oxidative damage [42]. Nrf2 is activated in healthy smokers, but its activation is impaired by oxidative stress in patients with COPD, resulting in reduced antioxidant gene expression [42]. Glutathione peroxidase is decreased in the lungs of COPD patients [43]. Glutathione peroxidase transgenic mice are protected against the development of inflammation and emphysema after cigarette smoke exposure, whereas glutathione peroxidase gene knockout increases the tissue destruction in the lung’s response to cigarette smoke [44]. Imbalance between oxidants and antioxidants probably plays an essential role in the chronic inflammation related to the pathology of COPD (Figure 1 and Figure 2). 

### 2.4. Reduced Responsiveness to Corticosteroids Caused by Oxidative Stress

Oxidative stress probably causes corticosteroid resistance in COPD. Oxidative stress reduces activity and expression of histone deacetylase-2 (HDAC2), which is required for inflammatory gene suppression [45], by activation of phosphoinositide-3-kinase (PI3K)-δ [46]. This phenomenon prevents the acetylation of glucocorticoid receptors, which is necessary for the inhibition of NF-κB that mediates the anti-inflammatory effects of corticosteroids, leading to reduced responsiveness to corticosteroids. Therefore, chronic lung inflammation is not fully inhibited by corticosteroids in COPD, different from mild asthma [6,33]. Recent preclinical studies have indicated that improvement of the redox balance by the administration of antioxidants or the stimulation of endogenous antioxidant response may overcome the corticosteroid resistance in COPD [47,48]. Nrf2 is known to act as an antioxidant. Sulforaphate, an activator of Nrf2, improves reduced responsiveness to corticosteroids mediated by upregulation of Nrf2 and enhancement of HDAC2 expression and activity in the allergen challenged mice that were exposed to cigarette smoke [49]. Nrf2 may be a potential molecular target for cigarette smoke-related resistance to corticosteroids in COPD. 

## 3. Dysfunction of Airway Smooth Muscle in COPD

### 3.1. Phenotype Changes

Airway smooth muscle cells in culture have the ability to change the degree of various functions such as contractility, proliferation, migration, and the synthesis of inflammatory mediators [50,51,52]. Alterations of airway smooth muscle cells from a contractile to a synthetic or a proliferative phenotype is involved in the pathophysiology of asthma and COPD, such as in airflow limitation, airway hyperresponsiveness, β_2_-adrenergic desensitization, and airway remodeling. These phenotype changes cause an abnormality in the function of airway smooth muscle. The dysfunction of airway smooth muscle occurs in asthma and COPD; and this phenomenon is associated with symptoms, a decline in lung function and the pathophysiology characterized by these diseases, and brings about contractile abnormality, release of inflammatory mediators, and hypertrophy in airway smooth muscle [53,54]. Airway smooth muscle cells can alter the degree of a variety of functions, including contraction, proliferation, migration, and the secretion of inflammatory mediators, referred to as phenotype plasticity. Characteristic features (major pathophysiology) of asthma and COPD, such as airflow limitation, airway hyperresponsiveness, β_2_-adrenergic desensitization, and airway remodeling, probably occur through phenotype changes in airway smooth muscle cells [50,51,52]. Changes between contractile and hyper-contractile, synthetic/proliferative phenotypes result from Ca^2+^ dynamics and Ca^2+^ sensitization, which are associated with the pathophysiology of these diseases [50,51,52]. Ca^2+^ dynamics through the large-conductance Ca^2+^-activated K^+^ (K_Ca_) channel/L-type voltage dependent Ca^2+^ (VDC) channel linkage, and Ca^2+^ sensitization through the RhoA (a monomeric G protein)/Rho-kinase (a target molecule of RhoA) pathway is involved not only in alterations in the contractile phenotype related to airflow limitation, airway hyperresponsiveness and β_2_-adrenergic desensitization but also in alteration of the synthetic/proliferative phenotype related to airway remodeling [50,52].

### 3.2. Airway Hyperresponsiveness

**Figure 3:**
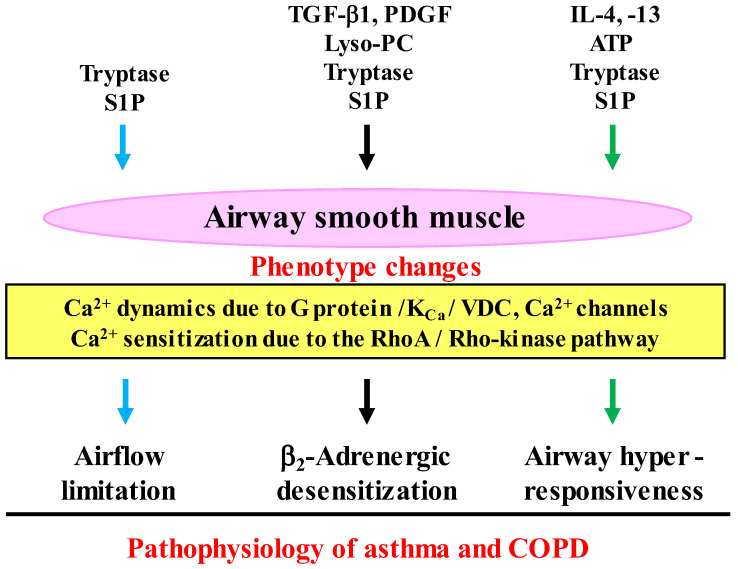
Interactions between inflammatory cells and airway smooth muscle cells in the pathophysiology of COPD. Functions (tension generation and response to contractile agents) of airway smooth muscle cells are altered (phenotype changes) by inflammatory substances (cytokines, growth factors, serine proteinases, phospholipids), which are synthesized in inflammatory cells (mast cells, eosinophils, etc.). Ca^2+^ signaling (Ca^2+^ dynamics and Ca^2+^ sensitization) is involved in the dysfunction of airway smooth muscle cells, leading to airflow limitation, β_2_-adrenergic desensitization and airway hyperresponsiveness (the pathophysiology features of asthma and COPD). Lyso-PC: lysophosphatidylcholine, TGF-β1: transforming growth factors-β1, PDGF: platelet-derived growth factor, ATP: adenosine triphosphate, S1P: sphingosine 1-phosphate, K_Ca_: Ca^2+^-activated K^+^ channel, VDC: L-type voltage-dependent Ca^2+^ channel. Arrows: activation.

Responsiveness to contractile and relaxant agents in airway smooth muscle is modified not only by inflammatory response related to asthma and COPD but also by excessive exposure to β_2_-adreneric agonists (Figure 3). Airway hyperresponsiveness is clinically shown as augmented responsiveness to muscarinic agonists or histamine. This pathophysiological alteration is a hallmark of asthma; but is observed in some cases of COPD [55]. Dysregulation of contractility is not as widely documented in airway smooth muscle of COPD, however patients with airflow limitation are shown to be very sensitive to inhaled methacholine [56], and the tissues of airway smooth muscle from patients with obstructive lung disease (with all but one characterized as having COPD) demonstrate significantly increased maximal isometric force and isometric stress, which is correlated to decline in lung function [57]. Increased contractility in airway smooth muscle causes manifestations (dyspnea, wheezing), airflow limitation and airway hyper-responsiveness. Airway hyperresponsiveness is associated with inflammation related to the pathogenesis of asthma and COPD. Airway hyperresponsiveness may have a harmful effect on therapy for asthma-COPD overlap, since response to inhaled corticosteroids with bronchodilators is reduced in patients with COPD who have eosinophilia and hyperresponsiveness in the airways [55]. 

Airway hyperresponsiveness is brought from Th2 cytokines such as interleukin (IL)-4, IL-13 that generate IgE [58,59]; involvement of IL-5 in this pathophysiology is still controversial. Adenosine triphosphate (ATP), which is released from injured epithelium in the airway by activated eosinophils, causes airway hyperresponsiveness with no change in concentration of intracellular Ca^2+^ [60] (Figure 3). Mast cells infiltrate airway smooth muscle in asthma, referred to as mast cell myositis. Exposure to tryptase and sphingosine 1-phosphate (S1P), which are released from mast cells, also causes airway smooth muscle contraction with an increase in concentration of intra-cellular Ca^2+^, and airway hyperresponsiveness with no change in Ca^2+^ (Figure 3) [61,62]. Ca^2+^ sensitization due to the RhoA/Rho-kinase processes is involved in airway hyperresponsiveness, which reflects a correlationship between inflammatory cells and airway smooth muscle cells [50,52,60,62,63].

### 3.3. β_2_-Adrenerigic Desensitization

Short- and long-acting β_2_-adrenergic agonists are widely used as bronchodilators as reliever and controller therapy for both asthma and COPD. Reduced responsiveness to β_2_-adrenergic agonists occurs in airway smooth muscle after excessive (repeated or sustained) exposure to these agonists and after persistent inflammation related to asthma and COPD, referred to as β_2_-adrenergic desensitization (tachyphylaxis) [64,65,66]. Repeated application of β_2_-adrenergic agonists results in a gradual reduction of their relaxant effects on muscarinic airway contraction. When isoprenaline, a full agonist, is repeatedly applied eight times every 30 min, relaxant effects of isoprenaline disappear almost completely in airway smooth muscle [66]. In contrast, relaxant effects of partial agonists such as formoterol are just slightly reduced under the same experimental conditions [67]. This phenomenon is not observed after excessive exposure to other cAMP-related agents bypassing β_2_-adrenergic receptors, such as forskolin or theophylline [64,65]. Agonist-induced dysfunction of β_2_-adrenergic receptors results from homologous desensitization in airway smooth muscle, not heterologous [64,65]. This phenomenon is caused by uncoupling the stimulatory G protein of adenylyl cyclase (G_s_) from phosphorylated β_2_-adrenergic receptors, not by down regulation, because of exposure time within 30 min [64,65]. 

Intrinsic efficacy, which is related to allosteric effects, is involved in homologous β_2_-adrenergic desensitization, which is associated with Ca^2+^ dynamics due to inhibitory linkage between G_s_ and large conductance Ca^2+^-activated K^+^ (K_Ca_) channels (Figure 3) [52,64,65,66,67,68]. The linkage of G_s_/K_Ca_ channels is deeply involved in the functional antagonism between muscarinic and β_2_-adrenergic action in airway smooth muscle [69,70,71]. Reduced responsiveness to β_2_-adrenergic agonists in airway smooth muscle also occur after persistent exposure to cytokines (IL-1β, tumor necrosis factor-α: TNF-α) [72], growth factors (Transforming Growth Factor-β1: TGF-β1, platelet-derived growth factor: PDGF) [73,74], phospholipids (S1P, lysophosphatidylcholine: Lyso-PC) [75,76], and mast cell tryptase [77], which are deeply involved in the pathogenesis of asthma and COPD (Figure 3). Pre-exposure to Lyso-PC, tryptase and S1P cause homologous β_2_-adrenergic desensitization mediated by Ca^2+^ sensitization due to RhoA/Rho-kinase processes. In contrast, pre-exposure to TGF-β1 and PDGF cause heterologous β_2_-adrenergic desensitization mediated by Ca^2+^ dynamics due to K_Ca_ channel inhibition. β_2_-Adrenergic desensitization in airway smooth muscle is caused not only by therapy but also pathogenesis related to asthma and COPD. 

### 3.4. Airway Remodeling

Airway smooth muscle can change to synthetic and proliferative phenotypes after exposure to various exogenous stimuli, such as matrix (ECM, in particular, collagen type 1 and fibronectin) and growth factors (PDGF and TGF-β), leading to airway remodeling [51,78]. Airway smooth muscle cells derived from patients with asthma show alterations towards a more proliferative phenotype than when derived from healthy subjects [79]. Exposure to IL-13 and PDGF-BB causes an increase in recapitulation of a more secretory and proliferative phenotype, resulted from a decrease in expression of the SR Ca^2+^ ATPase (a Ca^2+^ transporter) [80]. A synthetic phenotype is caused by an increase in synthetic organelles for protein and lipid synthesis (the Golgi apparatus and numerous mitochondria); and proliferative capacity is induced. Modulation towards proliferative and synthetic phenotypes is associated with an increase in non-muscle MHC, l-caldesmon, vimentin, α/β-PKC and CD44 homing cellular adhesion molecule [44]. In airway smooth muscle cell culture, 20–60% of the cells express secretory capacity; on the other hand, approximately 50% of the cells express proliferative capacity. Cytokine production and proliferation may be overlapping and not independent functions [81]. Hence, airway smooth muscle contributes to the inflammatory environments in both asthma and COPD, because of releasing various cytokines and chemokines in response to asthma and COPD related stimuli [82]. An increase in the amount of airway smooth muscle resulting from hypertrophy and hyperplasia is observed surrounding the central and peripheral airways in asthma and COPD (airway remodeling) [83,84]. 

## 4. Airway Smooth Muscles Regulated by Oxidative Stress

### 4.1. Expression of Oxidants in Airway Smooth Muscle

**Figure 4:**
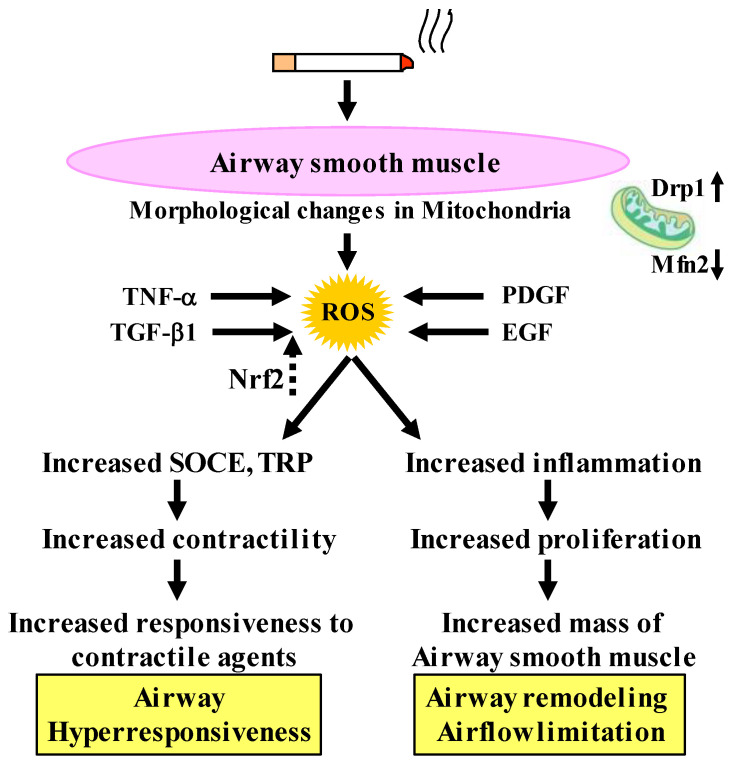
Involvement of oxidative stress in the dysfunctions of airway smooth muscle cells in COPD. Cigarette smoke enhances contractility caused by Ca^2+^ dynamics through TRP and SOCE. TNF-α and grows factors (TGF-β1, PDGF, and EGF) synthesize ROS, resulting in amplified cell proliferation through mitochondrial morphological changes, resulting in potentiated response to contractile agents (airway hyperresponsiveness) and increased mass of airway smooth muscle (airway remodeling, airflow limitation), which are pathological and pathophysiological characteristics of COPD. Nrf2 inhibits effects of TGF-β1 on oxidative stress. TRP: transient receptor potential, SOCE: store-operated Ca^2+^ entry. TNF-α: tumor necrosis factor-α, TGF-β1: transforming growth factor-β1, PDGF: platelet-derived growth factor, EGF: epidermal growth factor, Nrf2: nuclear erythroid-2 related factor 2, ROS: reactive oxygen species. Drp1: dynamin-related protein 1, Mfn2: mitofusin 2. Arrows: activation; dotted arrows: inactivation.

It is generally considered that oxidative stress influences the function of airway smooth muscle in COPD [85]. Cigarette smoke is the most encountered risk factor for COPD across the world. Cigarette smoke contributes to oxidative stress by induction of ROS production in COPD [86,87], leading to the development of COPD. Since airway smooth muscle plays an important role in tension and inflammation related to the pathophysiology of asthma and COPD, oxidative stress probably affects the function of airway smooth muscle in these diseases (Figure 4). Expression of the ROS generating enzyme NADPH oxidase (NOX)-4 is enhanced in airway smooth muscle from patients with COPD; increased expression of NOX-4 is correlated with disease severity and lung function decline [88]. H_2_O_2_ stimulated ROS production is completely abolished by an inhibition of NOX-4 in airway smooth muscle [89]. Therefore, oxidative stress causes the dysfunction of airway smooth muscle in COPD; and NOX-4 is probably a potential therapeutic target for COPD. Apocynin, a non-selective inhibitor of NOX, inhibits the inflammatory response to cigarette smoke in mice [90], although clinical trials have not been reported yet in COPD. 

### 4.2. Effects of Oxidative Stress on Contraction and Proliferation 

Oxidative stress can induce changes in the degree of various functions such as con-tractility, proliferation, migration, and the synthesis of inflammatory mediators in airway smooth muscle cell [50,51,52,67,85]. These phenotype changes of airway smooth muscle are deeply involved in the symptoms (e.g., dyspnea, wheezing) and the pathophysiology (e.g., airflow limitation, airway hyperresponsiveness, airway remodeling) of COPD (Figure 3 and Figure 4) [50,51,52,67,85]. It is unclear in detail whether cigarette smoke extract causes contraction of airway smooth muscle. However, H_2_O_2_ and 8-iso-PGF_2α_, which are oxidative stress markers that are elevated in exhaled breath (EB) from patients with COPD, cause contraction of tracheal smooth muscle in guinea pigs in a concentration-dependent manner [91,92], indicating that oxidative stress contributes to dyspnea, wheezing, and airflow limitation by an increase in the airway tension in COPD. Pre-exposure to cigarette smoke extract markedly enhances acetylcholine-induced force in human bronchial smooth muscle [93], indicating that cigarette smoke extract causes airway hyperresponsiveness. Nicotine, which can induce oxidative stress, also contributes to airway hyperresponsiveness (Figure 4) [94]. Nicotine-stimulated fibroblast-conditioned media increase expression of the contractile protein p-MLC in airway smooth muscle cells [95]; and nicotine also causes upregulation of nicotinic α7 acetyl-choline receptor (α7nAChR) expression in airway smooth muscle cells [94], indicating that nicotine enhances contractility in airway smooth muscle. TNF-α generates ROS in airway smooth muscle cells [96,97]; and TNF-α enhances contractile response to a muscarinic agonist (airway hyperresponsiveness) with ROS-dependent phosphorylation of MLC, which is the contractile protein [98].

Cigarette smoke increases cell numbers in bovine tracheal smooth muscle with cyclin D1 expression and DNA synthesis via activation of ERK 1/2 and p38 MAP kinase [99]. Exposure to cigarette smoke also causes cell proliferation on rat airway smooth muscle with expression of transient receptor potential cation channel subfamily M member 7 (TRPM7) which is activated by ROS [100,101]. Cigarette smoke causes mitochondrial fragmentation and disruption of mitochondrial networks through imbalance of the mitochondrial fission versus fusion. This phenomenon is due to an increase in dynamin-related protein 1 (Drp1) expression (fission) and a decrease in mitofusin 2 (Mfn2) expression (fusion), involving PI3K/Akt, PKC and ERK activation and transcriptional regulation via NF-κB [7]. This morphological alteration in mitochondria (the imbalance of fission/fusion) is associated with mitochondrial function, and is involved in proliferation/survival, response to inflammation, extracellular matrix production, and Ca^2+^ regulation in airway smooth muscle cells [102]. Cigarette smoke may induce not only a contractile phenotype but also a proliferative phenotype of airway smooth muscle cells, which may be associated with airflow limitation, airway hyperresponsiveness and airway remodeling in COPD (Figure 4). Therefore, oxidative stress probably causes the dysfunction (phenotype changes) of airway smooth muscle cells with changes in mitochondrial morphology [7]. Growth factors, such as TGF-β1, PDGF and EGF (epidermal growth factor), contribute to increased cell proliferation and dysfunction of airway smooth muscle with ROS production in COPD [85]. 

### 4.3. Inhibitory Effects of Antioxidants on Oxidative Stress Induced Proliferation

TGF-β1 can induce expression of the ROS generating enzyme NOX-4 in airway smooth muscle [37,99] via decapentaplegic family member 3 (SMAD3)/phosphoinositide 3-kinase (PI3K) signaling, leading to increased proliferation in airway smooth muscle cells [37,103]. This TGF-β1 induced dysfunction of airway smooth muscle cells is attenuated by activation of the antioxidant transcription factor Nrf2 [103]. In human tracheal smooth muscle cells, Nrf2 causes expression of the antioxidant genes heme-oxygenase 1 (HO-1), with activation of HO-1 reciprocally resulting in Nrf2 translocation from cytosol to nucleus [85]. The Nrf2/HO-1 signaling may potentially contribute to protecting against dysfunction of airway smooth muscle related to COPD since this process is involved in an inhibition not only of cell proliferation but also in inflammation and contraction of airway smooth muscle in the functional alterations caused by oxidative stress [85]. HO-1 activation causes an inhibition in TNF-α induced expression of intercellular adhesion molecule-1 (ICAM-1) and vascular cell adhesion molecule-1 (VCAM-1) and generation of IL-6 via suppression of TNF-α induced superoxide and H_2_O_2_ generation [104]. The Nrf2 pathway is implicated in protecting against airway hyperresponsiveness in animal models [105]. The expression of HO-1 is more decreased and net contractile moment is more increased in airway smooth muscle from Nrf2 knockout mice compared to airway smooth muscle from wild type mice, suggesting that the Nrf2/HO-1 process prevents augmented contractility by ROS production [106]. Therefore, the Nrf2/HO-1 process is probably a therapeutic target against the dysfunction of airway smooth muscle related to oxidative stress. 

### 4.4. Clinical Trials for Antioxidant Therapy 

Although it is well known that oxidative stress is a major factor leading to the development of COPD, specific therapy using antioxidants has not been established yet in this disease. Various oxidants are mentioned as a candidate involved in the pathophysiology of COPD. However, much remains unclear. Related oxidants may be different for each patient with COPD, and multiple oxidants may be involved for each patient. 

Dietary antioxidants (vitamin C, vitamin E, resveratrol, and flavonoids) dot not have significant effects on lung function and manifestation in patients with COPD [107,108]. Resveratrol may reduce ROS released from the airway epithelium in COPD in vitro; but the clinical relevance of this result is unclear [109]. A retrospective study has indicated that a Mediterranean diet that includes many dietary antioxidants may prevent the development of COPD [110]; but this result is still unclear because of confounding factors. 

In clinical studies using chemical agents, thiol-based antioxidants (*N*-acetylcysteine, carbocisteine, erdosteine) were administrated to patients with COPD. These agents that are used as expectorant to reduce mucus viscosity, act as antioxidants by elevating glutathione concentrations [111]. Some clinical studies on a small scale have indicated that these agents can decrease the number of exacerbations in COPD [112]. In contrast, large-scale trials have indicated that a high dose of *N*-Acetylcysteine has only modest efficacy in reducing exacerbation frequency [113], and that a low dose of that agent has no effect [114]. Nrf4 is generally considered to regulate multiple antioxidant genes. HO-1, which is an antioxidant gene regulated by Nrf2, can inhibit the development of emphysema, indicating that activation of Nrf2 may be effective in combatting oxidative stress in COPD. However, when sulforaphane, an activator of Nrf2, was administrated to patients with COPD for 4 weeks, this agent increased an antioxidant gene related to Nfr2 and inhibited oxidative stress and inflammation [115]. Clinical studies using antioxidants for COPD have not been reported other than these trials. Hence, the clinical significance of antioxidants still remains unclear in this disease. 

## 5. Calcium Signaling as Mechanisms of Oxidative Stress

### 5.1. Involvement of Ca^2+^ Dynamics in Oxidative Stress 

**Figure 5:**
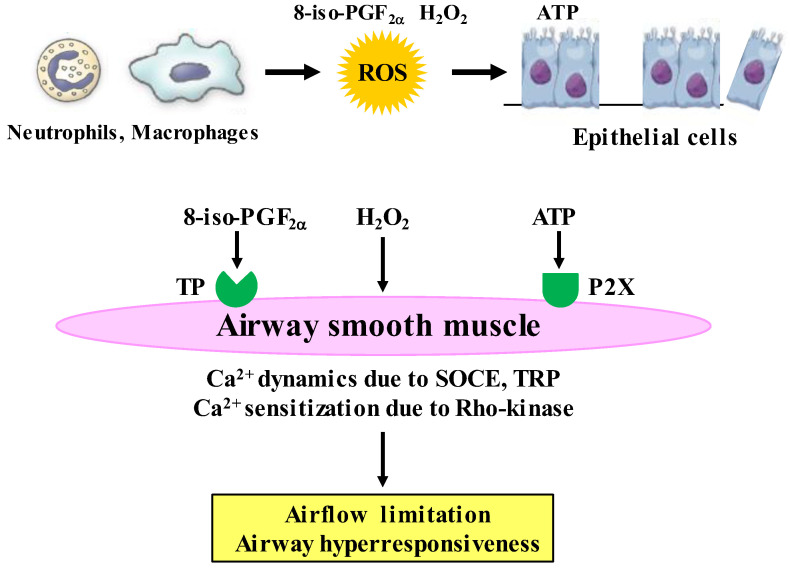
Roles of Ca^2+^ signaling in effects of oxidative stress related to COPD on airway smooth muscle. H_2_O_2_ and 8-iso-PGF_2α_, which are endogenous oxidants (oxidative stress biomarkers) synthesized in inflammatory cells, cause contraction with Ca^2+^ dynamics through Ca^2+^ channels and Ca^2+^ sensitization through the RhoA/Rho-kinase pathway. ATP, which is released from injury to airway epithelium caused by ROS, generates tension with Ca^2+^ dynamics through Ca^2+^ channels, and enhances muscarinic contraction with Ca^2+^ sensitization through the RhoA/Rho-kinase pathway. The Ca^2+^ signaling (Ca^2+^ dynamics and Ca^2+^ sensitization) may contribute to airflow limitation and airway hyperresponsiveness (pathophysiological features of COPD) caused by oxidative stress. ROS: reactive oxygen species, ATP: adenosine triphosphate, TP: thromboxane A_2_ receptors, P2X: P2X receptors (ATP-activated purinergic receptors), H_2_O_2_: hydrogen peroxide, 8-iso-PGF_2α_: 8-isoprostaglandin F_2α_.

**Figure 6:**
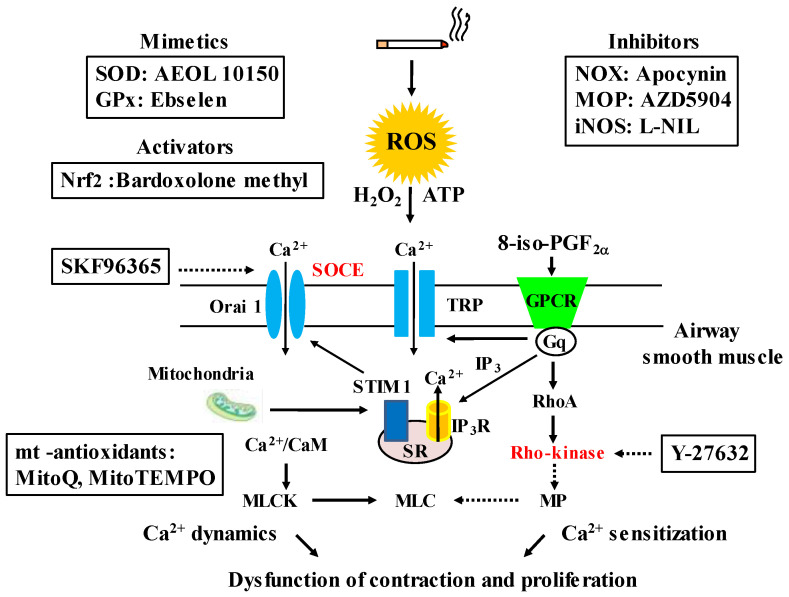
Chemical compounds that potentially act as antioxidants, and mechanisms (Ca^2+^ signaling) related to oxidative stress in COPD. The chemical compounds, which are mimetics of SOD, include: superoxide dismutase, GPx: glutathione peroxidase, activator of Nfr2: nuclear erythroid-2 related factor 2, inhibitors of NOX: NADPH oxidases, MOP: myeloperoxidase, iNOS: inducible nitric oxide synthase and mitochondria-related (mt)-related antioxidants; they are effective on oxidative stress related to COPD in animal models and vitro studies. Biomarkers of oxidative stress related to COPD (H_2_O_2_, 8-iso-PGF_2α_) and external ATP which are released from injured airway epithelium cause contraction of airway smooth muscle via Ca^2+^ dynamics due to SOCE and Ca^2+^ sensitization due to Rho-kinase. These Ca^2+^ signaling pathways are also associated with proliferation of airway smooth muscle cell, and are inhibited by SKF96365 and Y-27632, respectively. ROS: reactive oxygen species, TRP: transient receptor potential, SOCE: store-operated Ca^2+^ entry, STIM 1: stromal interaction molecule 1, CaM: calmodulin, MLCK: myosin light chain kinase: MP: myosin phosphatase, MLC: myosin light chain, IP3: inositol trisphosphate, IP_3_R: IP_3_ receptor, GPCR: G protein-coupled receptor. Arrows: activation; dotted arrows: inactivation.

It is considered that H_2_O_2_ and 8-isoprostane (8-iso-PG) F_2α_ could be useful biomarkers for oxidative stress in COPD [116,117], since these substances are elevated in exhaled breath from patients with COPD. To determine the intracellular mechanism underlining effects of oxidative stress on airway smooth muscle, H_2_O_2_, 8-iso-PG_2α_, and ATP were cumulatively applied to the fura-2 loaded tissues of guinea pig tracheal smooth muscle; and isometric tension and F_340_/F_380_ (an indicator for intracellular concentration of Ca^2+^) were simultaneously recorded. H_2_O_2_, 8-iso-PG_2α_, and ATP generated tension with an increase F_340_/F_380_ in a concentration-dependent manner (Figure 5) [91,92]. SKF96365, a non-selective inhibitor of Ca^2+^ channels, markedly inhibited tension induced by H_2_O_2_, 8-iso-PG_2α_, and ATP; in contrast, verapamil, an inhibitor of VDC channels, modestly inhibited them [91,92]. Since SKF96465 is an inhibitor of receptor-operated Ca^2+^ influx through transient receptor potential (TRP) channels and store-operated Ca^2+^ entry (SOCE) [118,119], Ca^2+^ dynamics due to TRP channels and/or SOCE are probably associated with this oxidant-induced contraction of tracheal smooth muscle; but, VDC channels may be less involved (Figure 4) [91,92]. When ligands connect to the GTP-binding (G) protein-coupled receptor (GPCR), Ca^2+^ is released from sarcoplasmic reticulum (SR), leading to SOCE, i.e., Ca^2+^ release-activated Ca^2+^ (CRAC) currents. SOCE is activated by stromal interaction molecule 1 (STIM 1), which is a Ca^2+^ sensor for store depletion in the SR (Figure 6). Although TRP channels may be related to the conduction of SOCE, it has recently been considered that the pore-forming protein Orai 1 is an essential component of the CRAC currents at the cell membrane [120] (Figure 6). This STIM 1/Orai 1 coupling contributes to SOCE in airway smooth muscle [121]. Although little is currently known about mechanisms of H_2_O_2_-induced contraction, the effect of 8-iso-PG_2α_ and ATP are associated with thromboxane A2 receptors (TP receptors) [92] and P2X (ATP-activated purinergic receptors) [60], respectively (Figure 5). Endogenous oxidants such as H_2_O_2_, 8-iso-PG_2α_, and ATP cause contraction of airway smooth muscle through Ca^2+^ dynamics, leading to dyspnea, wheezing and airflow limitation in COPD (Figure 5). The contractile effect of other oxidants in airway smooth muscle remains unclear. Although increased expression of NOX-4 is observed in airway smooth muscle from COPD patients [88], the potential involvement of NOX-4 in Ca^2+^ dynamics and contractility is still unknown. 

Recent studies using human airway smooth muscle cells have indicated that cigarette smoke and cigarette smoke extract cause Ca^2+^ influx through TRP ankyrin 1 (TRPA1) related to myosin light-chain phosphorylation, not L-type voltage-dependent Ca^2+^ (VDC) channels [122], and enhance Ca^2+^ influx in responses to bradykinin and histamine, which cause airway smooth muscle contraction through SOCE (Figure 4) [93]. Although cigarette smoke does not cause force generation in airway smooth muscle, cigarette smoke enhances response to bradykinin and histamine with SOCE (an increase in contractility), probably leading to airway hyperresponsiveness. Cigarette smoke contributes to dysfunction involved in contractility and proliferation in airway smooth muscle cells. Since cigarette smoke enhances not only Ca^2+^ influx through TRP channels and SOCE but also expression of Ca^2+^ regulatory proteins such as TRPC3, CD38, STIM1 (a sensor for Ca^2+^ concentration in the SR), and/or Orai1 (Ca^2+^ channels in the plasma membrane) in human airway smooth muscle cells, Ca^2+^ dynamics due to these processes play a critical role in alterations of function and structure in the airways mediated by smoking-related oxidation [123]. Expression of TRPC3 and CD38 is also markedly increased in the airways of patients with long-term smoking history compared to lifelong never smokers [122]. Mitochondria also act on regulation of cytosolic Ca^2+^ concentration through interaction between STIM1 and Orai1. Cigarette smoke-induced mitochondrial dysfunction enhances Ca^2+^ dynamics through SOCE [124] (Figure 6).

### 5.2. Involvement of Ca^2+^ Sensitization in Oxidative Stress 

On the other hand, Y-27632, an inhibitor of Rho-kinase, inhibits H_2_O_2_- and 8-iso-PG_2α_-induced contraction without a reduction in F_340_/F_380_ in the fura-2 loaded tracheal smooth muscle of guinea pigs [91,92]. These results have demonstrated that Ca^2+^ sensitization (increased sensitivity to intracellular Ca^2+^) related to Rho-kinase is involved in the airway tension induced by oxidant stress in COPD (Figure 5). When a contractile agonist connects to the GPCR, RhoA (a monomeric G protein) is activated by a trimeric G protein coupled to the GPCR. Rho-kinase, which is a target molecule of RhoA, inactivates myosin phosphatase acting on myosin phosphatase target subunit 1 (MYPT1) [62], leading to Ca^2+^-independent contraction (Ca^2+^ sensitization) in airway smooth muscle. Ca^2+^ sensitization related to the RhoA/Rho-kinase pathway contributes not only to muscarinic contraction [125] but also to augmented response to muscarinic agonists [60,62,63], indicating that this Ca^2+^ sensitization is probably associated with symptoms (dyspnea, wheezing) and the pathophysiology (airflow limitation, airway hyperresponsiveness) in COPD. It is still unclear how oxidative stress induces airway epithelial disorder; however, ATP may be released from injured epithelium leading to the pathogenesis of COPD [126]. Extra-cellular ATP causes a modest increase in F_340_/F_380_ without contractile response; and muscarinic contraction is significantly enhanced without an increase in F_340_/F_380_ after exposure to ATP (Figure 5) [60]. Extracellular ATP acts on P2X (ATP-activated purinergic receptors, different from GPCRs), and contributes to symptoms (dyspnea, wheezing) and the pathophysiology (airflow limitation, airway hyperresponsiveness) via Ca^2+^ sensitization related to the RhoA/Rho-kinase processes (Figure 5) [60]. Since 8-iso-PGF_2α_ acts on thromboxane receptors (GPCRs), oxidative stress due to 8-iso PGF_2α_ causes airway contraction via d a monomeric G protein. On the other hand, intracellular mechanisms are still unknown as regards Ca^2+^ sensitization by H_2_O_2_ and ATP. 

## 6. Toward the Progress of Therapy for COPD

**Figure 7:**
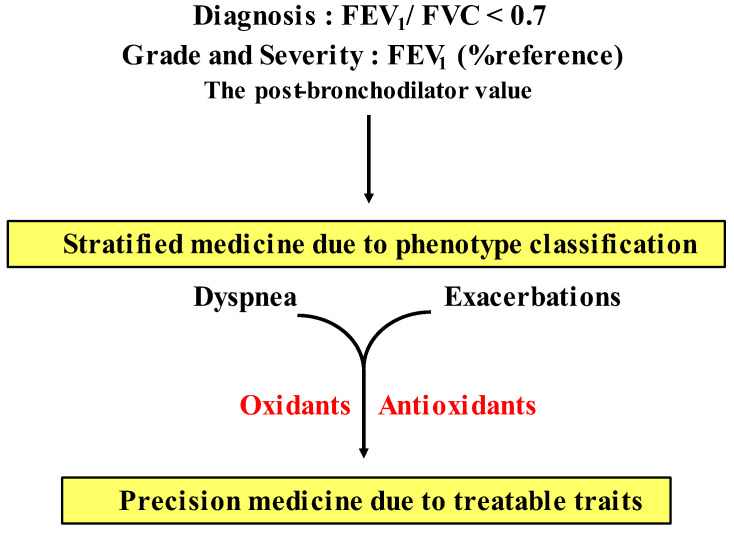
Precision medicine (personalized medicine) in COPD. The present guideline for COPD recommends that pharmacologic therapy for stable periods is carried out as a strategy based on symptoms (dyspnea) and frequency of exacerbations as treatable traits. Since COPD has heterogeneity, distinct phenotype classification is needed based on multidimensional approaches to advance from stratified medicine to personalized medicine in the management of COPD in near future. Although the clinical relevance of oxidative stress is still unclear, several oxidants can serve as treatable traits for development of precision medicine in COPD. See Section 6 in this text.

Previous clinical trials have shown that N-acetylcysteine (a thiol-based antioxidant, a precursor of glutathione) and sulforaphate (an activator of Nrf2) are not so effective in COPD, as noted above. In addition, previous studies using animal models and in vitro have indicated that several chemical compounds have effects against oxidants related to COPD, including: AEOL 10150 (a superoxide dismutase mimetics) [127], Ebselen (a glutathione peroxidase mimetic) [128], Apocynin (a NADPH oxidase inhibitor) [90], AZD 5904 (a myeloperoxidate inhibitor) [44], L-NIL (an: inducible nitric oxide synthase inhibitor) [129], Bardoxolone methyl (a Nrf2 activator) [115], mitoQ, mitoTEMPO (mitochondria-targeted antioxidants) [130,131,132] (Figure 6). However, clinical trials using them still have not been carried out. Effects of setanaxib (a NADPH oxidase inhibitor) on COPD have not been proved yet even in preclinical studies. H_2_O_2_ and 8-iso-PGF_2α_ cause contraction in airway smooth muscle; these contractile actions are attenuated in the presence of SKF96365 and Y-27632 in a concentration-dependent manner [91,92]. These chemical compounds are agents for Ca^2+^ signaling, i.e., the former is an inhibitor of Ca^2+^ influx such as SOCE and TRP channels (Ca^2+^ dynamics), and the latter is an inhibitor of the Rho-kinase (Ca^2+^ sensitization) (Figure 6). Since Ca^2+^ signaling due to Ca^2+^ dynamics and Ca^2+^ sensitization is probably related not only to contractile but also proliferative responses in COPD, these Ca^2+^ signaling-related molecules such as Orai 1, TRP and Rho-kinase, could be treatable traits for this disease. Clinical trials using chemical compounds related to Ca^2+^ signaling are needed to establish more suitable precision medicine (personalized medicine) for COPD (Figure 7).

Although many previous reports have indicated that oxidative stress is perhaps involved in the pathophysiology of COPD, little is currently known about its clinical relevance in detail, as described above. There is still uncertainty regarding which is the most important oxidant in COPD, or how many oxidants are involved in each patient with COPD. Involved oxidants may be different for each patient with COPD. In the present COPD guideline (the 2023 GOLD report), dyspnea and exacerbations are shown as treatable traits, and pharmacological therapy for stable COPD is recommended according to degree of dyspnea and frequency of exacerbation as current precision medicine (individualized medicine) [1]. However, since COPD has heterogeneity, the present strategy based on only these two treatable traits may be not sufficient to guide stable long-term management for COPD. Patients with COPD need to be classified according to distinct clinical phenotypes based on multiple dimensions (clinical, physiological, imaging, endotypes) [13]. Although H_2_O_2_ and 8-isoprostane, which are stated as biomarkers for oxidative stress in the 2023 GOLD report [1], are increased in the airway in most patients with COPD, novel precision medicine including these oxidants has not been established yet. To advance the management and treatment for COPD, it is necessary to provide precision medicine using oxidants and antioxidants as treatable traits based on stratification of patients according to oxidants and antioxidants for specific clinical phenotypes (Figure 6 and 7).

## 7. Conclusions

The inflammatory responses related to oxidative stress in COPD have great effects on the function of airway smooth muscle. Because of this interaction between inflammatory cells and airway smooth muscle cells, the function for contraction and proliferation is altered in airway smooth muscle cells, leading to symptoms (dyspnea, wheezing), and the pathophysiology (airflow limitation, airway hyperresponsiveness, airway remodeling) in COPD. Ca^2+^ signaling (Ca^2+^ dynamics and Ca^2+^ sensitization) is involved in the mechanisms of ROS (oxidative stress)-induced dysfunction of airway smooth muscle. Therefore, oxidative stress should be included in treatable traits for COPD to establish precision medicine; airway smooth muscle can be a novel therapeutic target for this disease. Research into Ca^2+^ signaling in airway smooth muscle will also be important for the development of a novel agent for COPD.

## References

[1] Global Initiative for Chronic Obstructive Lung Disease. Global Strategy for Prevention, Diagnosis and Management of Chronic Obstructive Pulmonary Disease: 2023 Report. https://goldcopd.org/.

[2] Hogg J.C., Timens W. (2009). The pathology of chronic obstructive pulmonary disease. Annu. Rev. Pathol..

[3] Barnes P.J., Burney P.G.J., Silverman E.K., Celli B.R., Vestbo J., Wedzicha J.A., Wouters E.F.M. (2015). Chronic obstructive pulmonary disease. Nat. Rev. Prim..

[4] Birnboim H.C. (1986). DNA strand breaks in human leukocytes induced by superoxide anion, hydrogen peroxide and tumor promoters are repaired slowly compared to breaks induced by ionizing radiation. Carcinogenesis.

[5] Agustí A., Hogg J.C. (2019). Update on the pathogenesis of chronic obstructive pulmonary disease. N. Engl. J. Med..

[6] Barnes P.J. (2022). Oxidative stress in chronic obstructive pulmonary disease. Antioxidants.

[7] Aravamudan B., Kiel A., Freeman M., Delmotte P., Thompson M., Vassallo R., Sieck G.C., Pabelick C.M., Prakash Y.S. (2014). Cigarette smoke-induced mitochondrial fragmentation and dysfunction in human airway smooth muscle. Am. J. Physiol. Lung Cell Mol. Physiol..

[8] Barnes P.J., Baker J., Donnelly L.E. (2019). Cellular senescence as a mechanism and target in chronic lung diseases. Am. J. Respir. Crit. Care Med..

[9] Tsuji T., Aoshiba K., Nagai A. (2004). Cigarette smoke induces senescence in alveolar epithelial cells. Am. J. Respir. Cell Mol. Biol..

[10] Childs B.G., Gluscevic M., Baker D.J., Laberge R.M., Marquess D., Dananberg J., van Deursen J.M. (2017). Senescent cells: An emerging target for diseases of ageing. Nat. Rev. Drug Discov..

[11] Kumar M., Seeger W., Voswinckel R. (2014). Senescence-associated secretory phenotype and its possible role in chronic obstructive pulmonary disease. Am. J. Respir. Cell Mol. Biol..

[12] Kirkham P.A., Barnes P.J. (2013). Oxidative stress in COPD. Chest.

[13] Agusti A., Bel E., Thomas M., Vogelmeier C., Brusselle G., Holgate S., Humbert M., Jones P., Gibson P.G., Vestbo J. (2016). Treatable traits: Toward precision medicine of chronic airway diseases. Eur. Respir. J..

[14] Domej W., Oettl K., Renner W. (2014). Oxidative stress and free radicals in COPD–Implications and relevance for treatment. Int. J. Chron. Obs. Pulmon. Dis..

[15] Di Stefano A., Caramori G., Oates T., Capelli A., Lusuardi M., Gnemmi I., Ioli F., Chung K.F., Donner C.F., Barnes P.J. (2002). Increased expression of nuclear factor-kappaB in bronchial biopsies from smokers and patients with COPD. Eur. Respir. J..

[16] Ravi A.K., Khurana S., Lemon J., Plumb J., Booth G., Healy L., Catley M., Vestbo J., Singh D. (2014). Increased levels of soluble interleu-kin-6 receptor and CCL3 in COPD sputum. Respir. Res..

[17] Shao M.X., Nakanaga T., Nadel J.A. (2004). Cigarette smoke induces MUC5AC mucin overproduction via tumor necrosis factor-α-converting enzyme in human airway epithelial (NCI-H292) cells. Am. J. Physiol. Lung Cell Mol. Physiol..

[18] van der Toorn M., Rezayat D., Kauffman H.F., Bakker S.J., Gans R.O., Koëter G.H., Choi A.M., van Oosterhout A.J., Slebos D.J. (2009). Lipid-soluble components in cigarette smoke induce mitochondrial production of reactive oxygen species in lung epithelial cells. Am. J. Physiol. Lung Cell. Mol. Physiol..

[19] Schumacker P.T., Gillespie M.N., Nakahira K., Choi A.M., Crouser E.D., Piantadosi C.A., Bhattacharya J. (2014). Mitochondria in lung biology and pathology: More than just a powerhouse. Am. J. Physiol. Lung Cell Mol. Physiol..

[20] Shapiro S.D. (2001). End-stage chronic obstructive pulmonary disease. Am. J. Respir. Crit. Care Med..

[21] Schaberg T., Klein U., Rau M., Eller J., Lode H. (1995). Subpopulations of alveolar macrophages in smokers and nonsmokers: Relation to the expression of CD11/CD18 molecules and superoxide anion production. Am. J. Respir. Crit. Care Med..

[22] Noguera A., Batle S., Miralles C., Iglesias J., Busquets X., MacNee W., Agustí A.G. (2001). Enhanced neutrophil response in chronic obstructive pulmonary disease. Thorax.

[23] Rahman I., van Schadewijk A.A., Crowther A.J., Hiemstra P.S., Stolk J., MacNee W., De Boer W.I. (2002). 4-Hydroxy-2-nonenal, a specific lipid peroxidation product, is elevated in lungs of patients with chronic obstructive pulmonary disease. Am. J. Respir. Crit. Care Med..

[24] Dekhuijzen P.N., Aben K.K., Dekker I., Aarts L.P., Wielders P.L., van Herwaarden C.L., Bast A. (1996). Increased exhalation of hydro-gen peroxide in patients with stable and unstable chronic obstructive pulmonary disease. Am. J. Respir. Crit. Care Med..

[25] Montuschi P., Collins J.V., Ciabattoni G., Lazzeri N., Corradi M., Kharitonov S.A., Barnes P.J. (2000). Exhaled 8-isoprostane as an in vivo biomarker of lung oxidative stress in patients with COPD and healthy smokers. Am. J. Respir. Crit. Care Med..

[26] Corradi M., Pignatti P., Manini P., Andreoli R., Goldoni M., Poppa M., Moscato G., Balbi B., Mutti A. (2004). Comparison between exhaled and sputum oxidative stress biomarkers in chronic airway inflammation. Eur. Respir. J..

[27] Bartoli M.L., Novelli F., Costa F., Malagrinò L., Melosini L., Bacci E., Cianchetti S., Dente F.L., Di Franco A., Vagaggini B. (2011). Malondialdehyde in exhaled breath condensate as a marker of oxidative stress in different pulmonary diseases. Mediat. Inflamm..

[28] Biernacki W.A., Kharitonov S.A., Barnes P.J. (2003). Increased leukotriene B4 and 8-isoprostane in exhaled breath condensate of patients with exacerbations of COPD. Thorax.

[29] Zhu A., Ge D., Zhang J., Teng Y., Yuan C., Huang M., Adcock I.M., Barnes P.J., Yao X. (2014). Sputum myeloperoxidase in chronic obstructive pulmonary disease. Eur. J. Med. Res..

[30] Antus B. (2016). Oxidative Stress Markers in Sputum. Oxid. Med. Cell Longev..

[31] Deslee G., Adair-Kirk T.L., Betsuyaku T., Woods J.C., Moore C.H., Gierada D.S., Conradi S.H., Atkinson J.J., Toennies H.M., Battaile J.T. (2010). Cigarette smoke induces nucleic-acid oxidation in lung fibroblasts. Am. J. Respir. Cell Mol. Biol..

[32] Thannickal V.J., Fanburg B.L. (2000). Reactive oxygen species in cell signaling. Am. J. Physiol. Lung Cell Mol Physiol..

[33] Barnes P.J. (2020). Oxidative stress-based therapeutics in COPD. Redox Biol..

[34] Osoata G.O., Hanazawa T., Brindicci C., Ito M., Barnes P.J., Kharitonov S., Ito K. (2009). Peroxynitrite elevation in exhaled breath condensate of COPD and its inhibition by fudosteine. Chest.

[35] O’Donnell C., Newbold P., White P., Thong B., Stone H., Stockley R.A. (2010). 3-Chlorotyrosine in sputum of COPD patients: Relation-ship with airway inflammation. COPD..

[36] Gorowiec M.R., Borthwick L.A., Parker S.M., Kirby J.A., Saretzki G.C., Fisher A.J. (2012). Free radical generation induces epithelial-to-mesenchymal transition in lung epithelium via a TGF-β1-dependent mechanism. Free Radic. Biol. Med..

[37] Michaeloudes C., Sukkar M.B., Khorasani N.M., Bhavsar P.K., Chung K.F. (2011). TGF-β regulates Nox4, MnSOD and catalase expres-sion, and IL-6 release in airway smooth muscle cells. Am. J. Physiol. Lung Cell Mol. Physiol..

[38] Drost E.M., Skwarski K.M., Sauleda J., Soler N., Roca J., Agusti A., MacNee W. (2005). Oxidative stress and airway inflammation in severe exacerbations of COPD. Thorax.

[39] Yao H., Arunachalam G., Hwang J.W., Chung S., Sundar I.K., Kinnula V.L., Crapo J.D., Rahman I. (2010). Extracellular superoxide dis-mutase protects against pulmonary emphysema by attenuating oxidative fragmentation of ECM. Proc. Natl. Acad. Sci. USA.

[40] Malhotra D., Thimmulappa R., Vij N., Navas-Acien A., Sussan T., Merali S., Zhang L., Kelsen S.G., Myers A., Wise R. (2009). Heightened endoplasmic reticulum stress in the lungs of patients with chronic obstructive pulmonary disease: The role of Nrf2-regulated proteasomal activity. Am. J. Respir. Crit. Care Med..

[41] Hwang J.W., Rajendrasozhan S., Yao H., Chung S., Sundar I.K., Huyck H.L., Pryhuber G.S., Kinnula V.L., Rahman I. (2011). FOXO3 deficiency leads to increased susceptibility to cigarette smoke-induced inflammation, airspace enlargement, and chronic obstructive pulmonary disease. J. Immunol..

[42] Liu Q., Gao Y., Ci X. (2019). Role of Nrf2 and Its Activators in Respiratory Diseases. Oxid. Med. Cell Longev..

[43] Vlahos R., Bozinovski S. (2013). Glutathione peroxidase-1 as a novel therapeutic target for COPD. Redox Rep..

[44] Geraghty P., Hardigan A.A., Wallace A.M., Mirochnitchenko O., Thankachen J., Arellanos L., Thompson V., D’Armiento J.M., Foronjy R.F. (2013). The glutathione peroxidase 1-protein tyrosine phosphatase 1B-protein phosphatase 2A axis. A key determinant of airway inflammation and alveolar destruction. Am. J. Respir. Cell Mol. Biol..

[45] Barnes P.J. (2013). Corticosteroid resistance in patients with asthma and chronic obstructive pulmonary disease. J. Allergy Clin. Immunol..

[46] To Y., Ito K., Kizawa Y., Failla M., Ito M., Kusama T., Elliott W.M., Hogg J.C., Adcock I.M., Barnes P.J. (2010). Targeting phosphoinositide-3-kinase-d with theophylline reverses corticosteroid insensitivity in chronic obstructive pulmonary disease. Am. J. Respir. Crit. Care Med..

[47] Lewis B.W., Ford M.L., Rogers L.K., Britt R.D. (2021). Oxidative stress promotes corticosteroid insensitivity in asthma and COPD. Antioxidants.

[48] Mei D., Tan W.S.D., Wong W.S.F. (2019). Pharmacological strategies to regain steroid sensitivity in severe asthma and COPD. Curr. Opin. Pharmacol..

[49] Sakurai H., Morishima Y., Ishii Y., Yoshida K., Nakajima M., Tsunoda Y., Hayashi S.Y., Kiwamoto T., Matsuno Y., Kawaguchi M. (2018). Sulforaphane ameliorates steroid insensitivity through an Nrf2-dependent pathway in cigarette smoke-exposed asthmatic mice. Free Radic. Biol. Med..

[50] Kume H. (2008). RhoA/Rho-kinase as a therapeutic target in asthma. Curr. Med. Chem..

[51] Wright D.B., Trian T., Siddiqui S., Pascoe C.D., Johnson J.R., Dekkers B.G., Dakshinamurti S., Bagchi R., Burgess J.K., Kanabar V. (2013). Phenotype modulation of airway smooth muscle in asthma. Pulm. Pharmacol. Ther..

[52] Kume H. (2021). Role of Airway Smooth Muscle in Inflammation Related to Asthma and COPD. Adv. Exp. Med. Biol..

[53] Yan F., Gao H., Zhao H., Bhatia M., Zeng Y. (2018). Roles of airway smooth muscle dysfunction in chronic obstructive pulmonary disease. J. Transl. Med..

[54] Camoretti-Mercado B., Lockey R.F. (2021). Airway smooth muscle pathophysiology in asthma. J. Allergy Clin. Immunol..

[55] Kume H., Hojo M., Hashimoto N. (2019). Eosinophil Inflammation and hyperresponsiveness in the airways as phenotypes of COPD, and usefulness of inhaled glucocorticosteroids. Front. Pharmacol..

[56] Ramsdell J.W., Nachtwey F.J., Moser K.M. (1982). Bronchial hyperreactivity in chronic obstructive bronchitis. Am. Rev. Respir. Dis..

[57] Opazo Saez A.M., Seow C.Y., Paré P.D. (2000). Peripheral airway smooth muscle mechanics in obstructive airways disease. Am. J. Respir. Crit. Care Med..

[58] Eum S.Y., Maghni K., Tolloczko B., Eidelman D.H., Martin J.G. (2005). IL-13 may mediate allergen-induced hyperresponsiveness inde-pendently of IL-5 or eotaxin by effects on airway smooth muscle. Am. J. Physiol. Lung Cell Mol. Physiol..

[59] Manson M.L., Säfholm J., James A., Johnsson A.K., Bergman P., Al-Ameri M., Orre A.C., Kärrman-Mårdh C., Dahlén S.E., Adner M. (2020). IL-13 and IL-4, but not IL-5 nor IL-17A, induce hyperresponsiveness in isolated human small airways. J. Allergy Clin. Immunol..

[60] Oguma T., Ito S., Kondo M., Makino Y., Shimokata K., Honjo H., Kamiya K., Kume H. (2007). Roles of P2X receptors and Ca^2+^ sensitization in extracellular adenosine triphosphate-induced hyperresponsiveness in airway smooth muscle. Clin. Exp. Allergy.

[61] Sekizawa K., Caughey G.H., Lazarus S.C., Gold W.M., Nadel J.A. (1989). Mast cell tryptase causes airway smooth muscle hyperresponsiveness in dogs. J. Clin. Invest..

[62] Kume H., Takeda N., Oguma T., Ito S., Kondo M., Ito Y., Shimokata K. (2007). Sphingosine 1-phosphate causes airway hyper-reactivity by Rho-mediated myosin phosphatase inactivation. J. Pharmacol. Exp. Ther..

[63] Taki F., Kume H., Kobayashi T., Ohta H., Aratake H., Shimokata K. (2007). Effects of Rho-kinase inactivation on eosinophilia and hyper-reactivity in murine airways by allergen challenges. Clin. Exp. Allergy.

[64] Kume H., Takagi K. (1997). Inhibitory effects of G_s_ on desensitization of β-adrenergic receptors in tracheal smooth muscle. Am. J. Physiol..

[65] Kume H., Takagi K. (1999). Inhibition of β-adrenergic desensitization by K_Ca_ channels in human trachealis. Am. J Respir. Crit. Care Med..

[66] Kume H., Ishikawa T., Oguma T., Ito S. (2003). Shimokata K, Kotlikoff MI. Involvement of Ca^2+^ mobilization in tachyphylaxis to β-adrenergic receptors in trachealis. Am. J. Respir. Cell Mol. Biol..

[67] Kume H., Fukunaga K., Oguma T. (2015). Research and development of bronchodilators for asthma and COPD with a focus on G protein/K_Ca_ channel linkage and β_2_-adrenergic intrinsic efficacy. Pharmacol. Ther..

[68] Kume H., Nishiyama O., Isoya T., Higashimoto Y., Tohda Y., Noda Y. (2018). Involvement of allosteric effect and K_Ca_ channels in crosstalk between β_2_-adrenergic and muscarinic M_2_ receptors in airway smooth muscle. Int. J. Mol. Sci..

[69] Kume H., Takai A., Tokuno H., Tomita T. (1989). Regulation of Ca^2+^-dependent K^+^-channel activity in tracheal myocytes by phos-phorylation. Nature.

[70] Kume H., Graziano M.P., Kotlikoff M.I. (1992). Stimulatory and inhibitory regulation of calcium-activated potassium channels by guanine nucleotide-binding proteins. Proc. Natl. Acad. Sci. USA.

[71] Kume H., Hall I.P., Washabau R.J., Takagi K., Kotlikoff M.I. (1994). β-Adrenergic agonists regulate K_Ca_ channels in airway smooth muscle by cAMP-dependent and -independent mechanisms. J. Clin. Invest..

[72] Guo M., Pascual R.M., Wang S., Fontana M.F., Valancius C.A., Panettieri R.A., Tilley S.L., Penn R.B. (2005). Cytokines regulate beta-_2_-adrenergic receptor responsiveness in airway smooth muscle via multiple PKA- and EP2 receptor-dependent mechanisms. Biochemistry.

[73] Ishikawa T., Kume H., Kondo M., Ito Y., Yamaki K., Shimokata K. (2003). Inhibitory effects of interferon-γ on the heterologous desensitization of β-adrenoceptors by transforming growth factor-β1 in tracheal smooth muscle. Clin. Exp. Allergy.

[74] Ikenouchi T., Kume H., Oguma T., Makino Y., Shiraki A., Ito Y., Shimokata K. (2008). Role of Ca^2+^ mobilization in desensitization of β-adrenoceptors by platelet-derived growth factor in airway smooth muscle. Eur. J. Pharmacol..

[75] Kume H., Ito S., Ito Y., Yamaki K. (2001). Role of lysophosphatidylcholine in the desensitization of β-adrenergic receptors by Ca^2+^ sensitization in tracheal smooth muscle. Am. J. Respir. Cell Mol. Biol..

[76] Makino Y., Kume H., Oguma T., Sugishita M., Shiraki A., Hasegawa Y., Honjo H., Kamiya K. (2012). Role of sphingosine-1-phosphate in β-adrenoceptor desensitization via Ca^2+^ sensitization in airway smooth muscle. Allergol. Int..

[77] Kobayashi M., Kume H., Oguma T., Makino Y., Ito Y., Shimokata K. (2008). Mast cell tryptase causes homologous desensitization of β-adrenoceptors by Ca^2+^ sensitization in tracheal smooth muscle. Clin. Exp. Allergy.

[78] Dekkers B.G., Schaafsma D., Nelemans S.A., Zaagsma J., Meurs H. (2007). Extracellular matrix proteins differentially regulate airway smooth muscle phenotype and function. Am. J. Physiol. Lung Cell Mol. Physiol..

[79] Johnson P.R., Roth M., Tamm M., Hughes M., Ge Q., King G., Burgess J.K., Black J.L. (2001). Airway smooth muscle cell proliferation is increased in asthma. Am. J. Respir. Crit. Care Med..

[80] Mahn K., Hirst S.J., Ying S., Holt M.R., Lavender P., Ojo O.O., Siew L., Simcock D.E., McVicker C.G., Kanabar V. (2009). Diminished sarco/endoplasmic reticulum Ca^2+^ ATPase (SERCA) expression contributes to airway remodeling in bronchial asthma. Proc. Natl. Acad. Sci. USA.

[81] Sukkar M.B., Stanley A.J., Blake A.E., Hodgkin P.D., Johnson P.R., Armour C.L., Hughes J.M. (2004). ‘Proliferative’ and ‘synthetic’ airway smooth muscle cells are overlapping populations. Immunol. Cell Biol..

[82] Prakash Y.S. (2013). Airway smooth muscle in airway reactivity and remodeling: What have we learned?. Am. J. Physiol. Lung Cell Mol. Physiol..

[83] James A.L., Elliot J.G., Jones R.L., Carroll M.L., Mauad T., Bai T.R., Abramson M.J., McKay K.O., Green F.H. (2012). Airway smooth muscle hypertrophy and hyperplasia in asthma. Am. J. Respir. Crit. Care Med..

[84] Jones R.L., Noble P.B., Elliot J.G., James A.L. (2016). Airway remodeling in COPD: It’s not asthma!. Respirology.

[85] Saunders R.M., Biddle M., Amrani Y., Brightling C.E. (2022). Stressed out—The role of oxidative stress in airway smooth muscle dysfunction in asthma and COPD. Free Radic. Biol. Med..

[86] Cheng S.E., Luo S.F., Jou M.J., Lin C.C., Kou Y.R., Lee I.T., Hsieh H.L., Yang C.M. (2009). Cigarette smoke extract induces cytosolic phospholipase A2 expression via NADPH oxidase, MAPKs, AP-1, and NF-kB in human tracheal smooth muscle cells. Free Radic. Biol. Med..

[87] Lin C.C., Lee I.T., Yang Y.L., Lee C.W., Kou Y.R., Yang C.M. (2010). Induction of COX-2/PGE2/IL-6 is crucial for cigarette smoke extract-induced airway inflammation: Role of TLR4-dependent NADPH oxidase activation. Free Radic. Biol. Med..

[88] Liu X., Hao B., Ma A., He J., Liu X., Chen J. (2016). The expression of NOX4 in smooth muscles of small airway correlates with the disease severity of COPD. Biomed Res. Int..

[89] Hollins F., Sutcliffe A., Gomez E., Berair R., Russell R., Szyndralewiez C., Saunders R., Brightling C. (2016). Airway smooth muscle NOX4 is upregulated and modulates ROS generation in COPD. Respir. Res..

[90] Oostwoud L.C., Gunasinghe P., Seow H.J., Ye J.M., Selemidis S., Bozinovski S., Vlahos R. (2016). Apocynin and ebselen reduce influenza A virus-induced lung inflammation in cigarette smoke-exposed mice. Sci. Rep..

[91] Kojima K., Kume H., Ito S., Oguma T., Shiraki A., Kondo M., Ito Y., Shimokata K. (2007). Direct effects of hydrogen peroxide on airway smooth muscle tone: Roles of Ca^2+^ influx and Rho-kinase. Eur. J. Pharmacol..

[92] Shiraki A., Kume H., Oguma T., Makino Y., Ito S., Shimokata K., Honjo H., Kamiya K. (2009). Role of Ca^2+^ mobilization and Ca^2+^ sensitization in 8-iso-PGF_2α_-induced contraction in airway smooth muscle. Clin. Exp. Allergy.

[93] Sathish V., Freeman M.R., Long E., Thompson M.A., Pabelick C.M., Prakash Y.S. (2015). Cigarette smoke and estrogen signaling in human airway smooth muscle. Cell Physiol. Biochem..

[94] Borkar N.A., Roos B., Prakash Y.S., Sathish V., Pabelick C.M. (2021). Nicotinic α7 acetylcholine receptor (α7nAChR) in human airway smooth muscle. Arch. Biochem. Biophys..

[95] Wongtrakool C., Grooms K., Bijli K.M., Crothers K., Fitzpatrick A.M., Hart C.M. (2014). Nicotine stimulates nerve growth factor in lung fibroblasts through an NFκB-dependent mechanism. PLoS ONE.

[96] Thabut G., El-Benna J., Samb A., Corda S., Megret J., Leseche G., Vicaut E., Aubier M., Boczkowski J. (2002). Tumor necrosis factor-alpha increases airway smooth muscle oxidants production through a NADPH oxidase-like system to enhance myosin light chain phosphorylation and contractility. J. Biol. Chem..

[97] Hsu C.K., Lee I.T., Lin C.C., Hsiao L.D., Yang C.M. (2014). Nox2/ROS-dependent human antigen R translocation contributes to TNF-α-induced SOCS-3 expression in human tracheal smooth muscle cells. Am. J. Physiol. Lung Cell Mol. Physiol..

[98] Okonski R., Zheng Y.M., Di Mise A., Wang Y.X. (2021). Reciprocal correlations of inflammatory and calcium signaling in asthma pathogenesis. Adv. Exp. Med. Biol..

[99] Pera T., Gosens R., Lesterhuis A.H., Sami R., van der Toorn M., Zaagsma J., Meurs H. (2010). Cigarette smoke and lipopolysaccharide induce a proliferative airway smooth muscle phenotype. Respir. Res..

[100] Lin X., Yang C., Huang L., Chen M., Shi J., Ouyang L., Tang T., Zhang W., Li Y., Liang R. (2016). Upregulation of TRPM7 augments cell proliferation and interleukin-8 release in airway smooth muscle cells of rats exposed to cigarette smoke. Mol. Med. Rep..

[101] Abiria S.A., Krapivinsky G., Sah R., Santa-Cruz A.G., Chaudhuri D., Zhang J., Adstamongkonkul P., DeCaen P.G., Clapham D.E. (2017). TRPM7 senses oxidative stress to release Zn^2+^ from unique intracellular vesicles. Proc. Natl. Acad. Sci. USA.

[102] Aravamudan B., Thompson M., Sieck G.C., Vassallo R., Pabelick C.M., Prakash Y.S. (2017). Functional effects of cigarette smoke-induced changes in airway smooth muscle mitochondrial morphology. J. Cell Physiol..

[103] Sturrock A., Huecksteadt T.P., Norman K., Sanders K., Murphy T.M., Chitano P., Wilson K., Hoidal J.R., Kennedy T.P. (2007). Nox4 mediates TGF-β1-induced retinoblastoma protein phosphorylation, proliferation, and hypertrophy in human airway smooth muscle cells. Am. J. Physiol. Lung Cell Mol. Physiol..

[104] Lee I.T., Luo S.F., Lee C.W., Wang S.W., Lin C.C., Chang C.C., Chen Y.L., Chau L.Y., Yang C.M. (2009). Overexpression of HO-1 protects against TNF-alpha-mediated airway inflammation by down-regulation of TNFR1-dependent oxidative stress. Am. J. Pathol..

[105] Rangasamy T., Guo J., Mitzner W.A., Roman J., Singh A., Fryer A.D., Yamamoto M., Kensler T.W., Tuder R.M., Georas S.N. (2005). Disruption of Nrf2 enhances susceptibility to severe airway inflammation and asthma in mice. J. Exp. Med..

[106] An S.S., Kim J., Ahn K., Trepat X., Drake K.J., Kumar S., Ling G., Purington C., Rangasamy T., Kensler T.W. (2009). Cell stiffness, contractile stress and the role of extracellular matrix. Biochem. Biophys. Res. Commun..

[107] Tsiligianni I.G., van der Molen T. (2010). A systematic review of the role of vitamin insufficiencies and supplementation in COPD. Respir. Res..

[108] Biswas S., Hwang J.W., Kirkham P.A., Rahman I. (2013). Pharmacological and dietary antioxidant therapies for chronic obstructive pulmonary disease. Curr. Med. Chem..

[109] Culpitt S.V., Rogers D.F., Fenwick P.S., Shah P., De Matos C., Russell R.E., Barnes P.J., Donnelly L.E. (2003). Inhibition by red wine extract, resveratrol, of cytokine release by alveolar macrophages in COPD. Thorax.

[110] Fischer A., Johansson I., Blomberg A., Sundström B. (2019). Adherence to a Mediterranean-like diet as a protective factor against COPD: A Nested Case-Control Study. COPD.

[111] Biswas S.K., Rahman I. (2009). Environmental toxicity, redox signaling and lung inflammation: The role of glutathione. Mol. Asp. Med..

[112] Grandjean E.M., Berthet P., Ruffmann R., Leuenberger P. (2000). Efficacy of oral long-term N-acetylcysteine in chronic bronchopul-monary disease: A meta-analysis of published double-blind, placebo-controlled clinical trials. Clin. Ther..

[113] Zheng J.P., Wen F.Q., Bai C.X., Wan H.Y., Kang J., Chen P., Yao W.Z., Ma L.J., Li X., Raiteri L. (2014). PANTHEON study group. Twice daily N-acetylcysteine 600 mg for exacerbations of chronic obstructive pulmonary disease (PANTHEON): A randomised, double-blind placebo-controlled trial. Lancet Respir. Med..

[114] Decramer M., Rutten-van Mölken M., Dekhuijzen P.N., Troosters T., van Herwaarden C., Pellegrino R., van Schayck C.P., Olivieri D., Del Donno M., De Backer W. (2005). Effects of N-acetylcysteine on outcomes in chronic obstructive pul-monary disease (Bronchitis Randomized on NAC Cost-Utility Study, BRONCUS): A randomised placebo-controlled trial. Lancet.

[115] Sussan T.E., Rangasamy T., Blake D.J., Malhotra D., El-Haddad H., Bedja D., Yates M.S., Kombairaju P., Yamamoto M., Liby K.T. (2009). Targeting Nrf2 with the triterpenoid CDDO-imidazolide attenuates cigarette smoke-induced emphysema and cardiac dysfunction in mice. Proc. Natl. Acad. Sci. USA.

[116] Repine J.E., Bast A., Lankhorst I. (1997). Oxidative stress in chronic obstructive pulmonary disease. Oxidative Stress Study Group. Am. J. Respir. Crit. Care Med..

[117] Praticò D., Basili S., Vieri M., Cordova C., Violi F., Fitzgerald G.A. (1998). Chronic obstructive pulmonary disease is associated with an increase in urinary levels of isoprostane F_2α_-III, an index of oxidant stress. Am. J. Respir. Crit. Care Med..

[118] Thebault S., Roudbaraki M., Sydorenko V., Shuba Y., Lemonnier L., Slomianny C., Dewailly E., Bonnal J.L., Mauroy B., Skryma R. (2003). α_1_-Adrenergic receptors activate Ca^2+^-permeable cationic channels in prostate cancer epithelial cells. J. Clin. Invest..

[119] Wang J., Shimoda L.A., Sylvester J.T. (2004). Capacitative calcium entry and TRPC channel proteins are expressed in rat distal pulmonary arterial smooth muscle. Am. J. Physiol. Lung Cell Mol. Physiol..

[120] Prakriya M., Feske S., Gwack Y., Srikanth S., Rao A., Hogan P.G. (2006). Orai1 is an essential pore subunit of the CRAC channel. Nature.

[121] Spinelli A.M., González-Cobos J.C., Zhang X., Motiani R.K., Rowan S., Zhang W., Garrett J., Vincent P.A., Matrougui K., Singer H.A. (2012). Airway smooth muscle STIM1 and Orai1 are upregulated in asthmatic mice and mediate PDGF-activated SOCE, CRAC currents, proliferation, and migration. Pflugers Arch..

[122] Jiang Q., Fu X., Tian L., Chen Y., Yang K., Chen X., Zhang J., Lu W., Wang J. (2014). NOX4 mediates BMP4-induced upregulation of TRPC1 and 6 protein expressions in distal pulmonary arterial smooth muscle cells. PLoS ONE.

[123] Lin J., Taggart M., Borthwick L., Fisher A., Brodlie M., Sassano M.F., Tarran R., Gray M.A. (2021). Acute cigarette smoke or extract ex-posure rapidly activates TRPA1-mediated calcium influx in primary human airway smooth muscle cells. Sci. Rep..

[124] Wylam M.E., Sathish V., VanOosten S.K., Freeman M., Burkholder D., Thompson M.A., Pabelick C.M., Prakash Y.S. (2015). Mechanisms of cigarette smoke effects on human airway smooth muscle. PLoS ONE.

[125] Prakash Y.S., Pabelick C.M., Sieck G.C. (2017). Mitochondrial dysfunction in airway disease. Chest.

[126] Ito S., Kume H., Honjo H., Katoh H., Kodama I., Yamaki K., Hayashi H. (2001). Possible involvement of Rho kinase in Ca^2+^ sensitization and mobilization by MCh in tracheal smooth muscle. Am. J. Physiol. Lung Cell Mol. Physiol..

[127] Mortaz E., Folkerts G., Nijkamp F.P., Henricks P.A. (2010). ATP and the pathogenesis of COPD. Eur. J. Pharmacol..

[128] Smith K.R., Uyeminami D.L., Kodavanti U.P., Crapo J.D., Chang L.Y., Pinkerton K.E. (2002). Inhibition of tobacco smoke-induced lung inflammation by a catalytic antioxidant. Free Rad. Biol. Med..

[129] Churg A., Marshall C.V., Sin D.D., Bolton S., Zhou S., Thain K., Cadogan E.B., Maltby J., Soars M.G., Mallinder P.R. (2012). Late intervention with a myeloperoxidase inhibitor stops progression of experimental COPD. Am. J. Respir. Crit. Care Med..

[130] Seimetz M., Parajuli N., Pichl A., Veit F., Kwapiszewska G., Weisel F.C., Milger K., Egemnazarov B., Turowska A., Fuchs B. (2011). Inducible NOS inhibition reverses tobacco smoke-induced emphysema and pulmonary hypertension in mice. Cell.

[131] Wiegman C.H., Michaeloudes C., Haji G., Narang P., Clarke C.J., Russell K.E., Bao W., Pavlidis S., Barnes P.J., Kanerva J. (2015). Oxidative stress-induced mitochondrial dysfunction drives inflammation and airway smooth muscle remodeling in patients with chronic obstructive pulmonary disease. J. Allergy Clin. Immunol..

[132] Hara H., Araya J., Ito S., Kobayashi K., Takasaka N., Yoshii Y., Wakui H., Kojima J., Shimizu K., Numata T. (2013). Mi-tochondrial fragmentation in cigarette smoke-induced bronchial epithelial cell senescence. Am. J. Physiol. Lung Cell Mol. Physiol..

